# Long term remission of metastatic placental site trophoblastic tumor (PSTT): Case report and review of literature

**DOI:** 10.1186/1477-7819-3-34

**Published:** 2005-06-15

**Authors:** Nadereh Behtash, Fatemeh Ghaemmaghami, Malihe Hasanzadeh

**Affiliations:** 1 Gynecology Oncology, Tehran University of Medical Sciences, Tehran, Iran

## Abstract

**Background:**

Placental site trophoblastic tumor (PSTT) is a rare and unique form of gestational trophoblastic disease (GTD). This tumor represents a neoplastic transformation of intermediate trophoblastic cells. We document a case of long term remission in a patient with metastatic PSTT.

**Case presentaion:**

A 27-year-old patient with metastatic PSTT was treated with combination therapy (chemotherapy and surgery). Patient is alive after 10 years without any evidence of recurrence. Literature on PSTT was searched using Medline and cross references, and pertinent articles were reviewed.

**Conclusion:**

With surgery and chemotherapy it is possible to achieve long-term remission in metastatic PSTT. Only a handful of previously reported cases with prolonged remission had been treated with the described combined chemotherapy and surgical approach. We suggest that this approach may be recommended for metastatic PSTT.

## Background

Placental Site Trophoblastic Tumor (PSTT) is a rare form of gestational trophoblastic disease (GTD) and was described by Kurman *et al *[1]. In 1976, it was thought to be an exaggerated expression of the invasive nature of normal trophoblastic tissue which did not assume the characteristics of a malignant tumor. Twigg's *et al*, described a patient with trophoblastic pseudotumor which was progressive and fatal in 1981 [2]. Scully and Young in 1981 described additional 14 cases of which two died from metastatic disease [3].

Pathologically, the tumor is characterized by mononuclear intermediate trophoblastic cells with occasional multinuclear intermediate trophoblastic cells and occasional multinuclear giant cell which infiltrate both myometrium and blood vessels [4]. Immunohistochemical staining reveals many prolactin cells and few gonadotropin producing ones. Thus gonadotropin levels may be normal to elevated [5].

The etiology, epidemiology and risk-factors of PSTT are poorly understood. Presenting symptoms generally include irregular bleeding or amenorrhea and rarely nephrotic syndrome, sepsis, and erythrocytosis [6], or the metastatic sites may be the presenting symptoms. PSTT presents with metastases in about 10% of the cases [7] and metastases develop in an additional 10% during follow-up [8].

Although the majority of patients with non metastatic PSTT are cured by hysterectomy, a number of cases require aggressive treatment with chemotherapy and/or radiation.

This paper presents one case of PSTT treated by combined treatment modalities. Hysterectomy was performed in this case at a time when the exact diagnosis was not apparent.

We also reviewed the literature for the different strategies in treatment of the metastatic disease.

## Case Presentation

In Sep. 1994, a 27-year-old woman (gravida 3, para 3) was referred to Gynecology Oncology Unit in Vali-e-Asr Hospital, Tehran, Iran. Her previous pregnancy was terminated at 40 weeks, with vaginal delivery in May 1991. After a few months of abnormal uterine bleeding, she underwent dilatation and curettage and then a total abdominal hysterectomy (2 months before referral). Histology of the resected specimen revealed choriocarcinoma.

**Figure 1:**
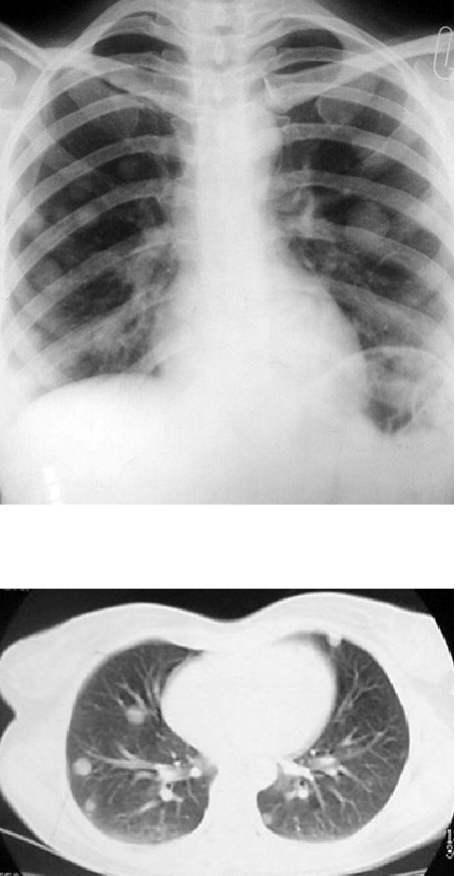
Chest X-ray and Computerized tomographic scan showing multiple metastatic nodules in the lung

**Figure 2:**
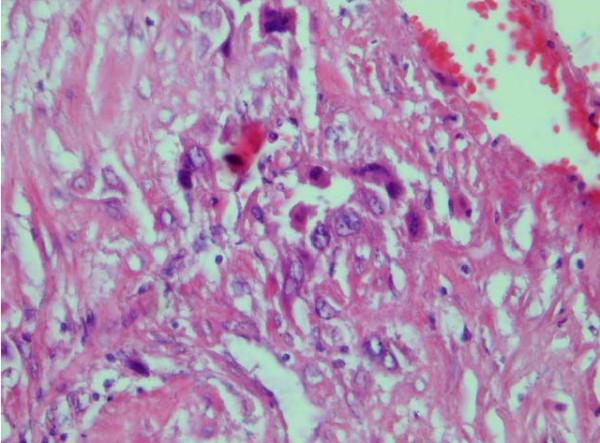
Photomicrograph showing proliferating intermediate trophoblast with scares cytotophoblastic and systrophoblastic elements

Upon her first visit to our clinic, she was in respiratory distress, and had cough with bloody sputum. Physical examination revealed no abnormal finding except wheezing. Chest X-ray and computerized tomographic (CT) scan showed multiple metastatic nodules in both lungs parenchyma (Figure 1). Abdominal and pelvic CT was normal. Histology was reviewed by two expert pathologists in our center, and PSTT was confirmed (Figure 2). Serum β-hCG level was 210 mIU/ml. Serial serum β-hCG ranged between 170 – 250, and increased after 3 courses of combination chemotherapy with methotrexate, actinomycin and cyclophosphamide.

Three years later, in november 1994, the patient was given three cycles of EMA chemotherapy. However, due to plateau titer of β-hCG the chemotherapy regimen was changed to EMA-CE. Reassessment of staging work-up was performed using pelvic ultrasonography that revealed a hypoechoic mass in right ovary (42 mm). At the end of six courses of EMA-CE chemotherapy, serum β-hCG remained at 50 mIu/ml.

In May 1995, patient underwent laparotomy and right oophrectomy one month following which the serum β-hCG was negative. Patient is on regular follow-up (clinical exam, β-hCG tests, pelvic and abdominal sonography and chest CT) and has shown no signs of local or systemic recurrence.

## Discussion

PSTT is a rare form of GTD with unpredictable malignant potential and highly variable clinical course [9]. It could also present as a fulminant metastatic disease, resistant to conventional treating modalities. PSTT accounts for 3.1/1000 to 2/100 of all trophoblastic diseases [10,11]. Rate of PSTT to choriocarcinoma has been reported to be 1/138 [10]. The disease is usually seen in young women, although cases have been reported in post-menopausal women as well. The mean age at diagnosis is 31 to 33 years, and it can appear following any type of pregnancy [10-12]. The antecedent pregnancy is a full term normal in 53% of the cases [11,12], or a molar pregnancy seen in 21% of the cases [11,13]. The mean interval from the last pregnancy and diagnosis of PSTT can vary from several weeks up to 15 years [14].

Unlike choriocarcinoma, the level of serum β-hCG in PSTT correlate neither with tumor burden, nor with the malignant behavior. Thus β-hCG appears to have no predictive value and the disease may still progress even if levels are not raised [14,23]. The range of serum β-hCG concentrations at diagnosis in 79% of the patients is below 1000 and in 58% lower than 500 [11].

In NETDC report, most patients (12 from 13 patients) had β-hCG levels under 500 mIU/ml [12]. PSTT clearly present a wide spectrum of clinical course. The most frequent presenting symptom is vaginal bleeding (79%) [11]. Ninty-two percent of Feltmate series presented with amenorrhea or abnormal bleeding [12].

Outcome of PSTT as reported in literature is highly variable [9]. All cases of metastasis to vital organs, such as the brain, result in mortality despite all forms of treatment.

PSTT is usually confined to uterus at the time of diagnosis. In cases with distant spread of disease, metastases predominantly occur in the vagina and in lung as in our case. Brain metastases have been detected in more than half of PSTT patients [17,18]. Lung metastases have been reported in nearly one third of the patients in Charing Cross series [11]. In this series all the seven deaths in patients with lung metastasis occurred in patients that presented 4 years or more after last pregnancy [11].

Extrauterine spread of the disease appears to be the most useful prognostic factor for progression [19,20]. The interval from the last known antecedent pregnancy appears to be a second major prognostic variable in PSTT. In a multivariate analysis, the risk for unfavorable behavior of the disease increased considerably with the length of this interval [9,21]. Diagnosis less than 2 years from the antecedent pregnancy, and the disease localized to the uterus are associated with better outcome [9,11]. How *et al*, found that likelihood for fatal outcome was 14 times higher if the mitotic count was well above 5 [20].

In contrast to choriocarcinoma, PSTT is relatively resistant to chemotherapy. Consequently surgery is the mainstay of treatment. In most series more than one treatment modalities have been used [9,11,14,19].

Conservative surgery in form of dilatation and curettage is justified only if the fertility is to be preserved [22]. Further, as PSTT is generally resistant to chemotherapy, there are only few long term survivors, with metastatic PSTT despite intensive multimodal therapy [9,23]. Local uterine resection may be considered in such patients and in those who wants to retain fertility. When local resection is considered, ultrasound, MRI, and/or PET scan may identify the site of residual tumor.

In other cases, hysterectomy followed by adjuvant chemotherapy is primary treatment- chemotherapy within one week of hysterectomy, results in lower recurrence rates [14]. The most recent data from different centers, suggest that EMA/EP is the most effective treatment for metastatic or recurrent PSTT [9].

## Conclusion

PSTT is a disorder of uncertain outcome, some of the patients do well while others have poor outcome. Combined treatment have been reported in 26% – 55% of the cases. Second look surgery, and multiple courses of combined chemotherapy may be necessary for remission.

## Competing interests

The author(s) declare that they have no competing interest

## Authors' contributions

**NB **carried out the surgery and participated in drafting the manuscript.

**FG **carried out the chemotherapy and follow-ups.

**MH **participated in the design of the study and helped to draft the manuscript. All authors read and approved the final manuscript.
